# Use of telehealth for facilitating the diagnostic assessment of Autism Spectrum Disorder (ASD): A scoping review

**DOI:** 10.1371/journal.pone.0236415

**Published:** 2020-07-23

**Authors:** Manahil Alfuraydan, Jodie Croxall, Lisa Hurt, Mike Kerr, Sinead Brophy

**Affiliations:** 1 School of Medicine, Swansea University, Swansea, United Kingdom; 2 College of Applied Medical Sciences, King Faisal University, Hofuf, Saudi Arabia; 3 Division of Population Medicine, School of Medicine, Cardiff University, Cardiff, United Kingdom; 4 Division of Psychological Medicine and Clinical Neuroscience, Cardiff University, Cardiff, United Kingdom; 5 National Centre for Population Health and Wellbeing, School of Medicine, Swansea University, Swansea, United Kingdom; University of Wollongong, AUSTRALIA

## Abstract

There is a significant delay between seeking help and a confirmed diagnosis of Autism Spectrum Disorder (ASD). This delay can lead to poor outcomes for both the families and individuals. Telehealth potentially offers a way of improving the diagnostic pathway for ASD. We conducted a scoping review examining which telehealth approaches are used in the diagnosis and assessment of ASD in children and adults, whether they are feasible and acceptable, and how they compare with face-to-face diagnosis and assessment methods. A search for all peer-reviewed articles, combining the terms of autism and telehealth was conducted from 2000 to 2019. A total of 10 studies were identified for inclusion in the review. This review of the literature found there to be two methods of using telehealth: (a) Real-Time method e.g. video conferencing that enables teams in different areas to consult with the families and to assess the child/adult in real time and (b) A Store-and-Forward method as Naturalistic Observation Diagnostic Assessment (NODA) system to upload videos of child’s behaviors to a webportal that enables the clinicians to make an assessment remotely. The findings were positive, finding there to be high agreement in terms of the diagnosis between remote methods and face to face methods and with high levels of satisfaction among the families and clinicians. This field is in the very early stages and so only studies with small sample size using surveys and interviews were identified but the findings suggest that there is potential for telehealth methods to improve access to assessment and diagnosis of ASD used in conjunction with existing methods, especially for those with clear autism traits and adults with ASD. Larger randomised controlled trials of this technology are warranted.

## Introduction

Autism Spectrum Disorder (ASD) is a developmental condition characterised by impairment in terms of social communication and social interaction and a repetitive and restricted pattern of interest, behaviour and activity [1]. Around 1 in 54 children in the United States has been diagnosed with ASD [2]. In respect of the diagnostic criteria, the latest revision of the Diagnostic and Statistical Manual of Mental Disorders “DSM-5”, published in May 2013, approved the umbrella term ASD without any subtypes. It organised the impairments into two domains: difficulties in social communication and social interaction; and restricted and repetitive behaviour, interests, or activities [1]. The diagnosis of ASD may include physical examinations to rule out physical causes of difficulties, speech and language assessment, observation, and a history of the child’s development and behaviour, focusing on features consistent with the DSM-5 criteria [3, 4]. Commonly used tools to aid diagnosis include the Autism Diagnostic Interview–Revised (ADI-R) and Autism Diagnostic Observation Schedule (ADOS-2) [4]. The ADI-R is a semi-structured interview that is conducted by clinician with a parent/carer who is familiar with developmental history as well as child’ current behaviour [5]. The ADOS-2 has 5 modules which involve 40–60 minute protocols of activities that are based on playing, communication and social interactions [6].

Research reports a delay of approximately 20 months to 5 years between seeking help and a confirmed diagnosis of ASD [7–10]. This may result in delayed access to early intervention services, which are critical for positive outcomes [11–15]. The long wait times also cause stress to individuals and families [7, 16]. Some of the reasons for the delays in diagnosis include the shortage of healthcare professionals [17] and several specialist and treatment appointments are often needed to make a diagnosis. Multiple meetings with a variety of professionals in different locations can also be very stressful for individuals who might later be diagnosed with ASD. This is as people with ASD are very sensitive to changes in their situations and excessively reliant on routines [1]. Some families have to travel long distances to access these services [18]. This is not only a cost to the family but also to the specialist healthcare teams who travel to reach different areas. Recent research found that healthcare and educational systems work independent from each other with little crossover of activity [19], therefore it may be difficult to get multi-disciplinary teams together, especially when they span different disciplines.

Overall, there seems to be some evidence to indicate that parents of children with ASD and adults face difficulties in getting ASD diagnosis. Therefore, more information is needed as to how to improve diagnostic services to address the well-being of the child and improve the experiences of the parents and adults with ASD. Telehealth can provide help and support to populations with specific health and well-being needs. Telehealth is identified as a “mechanism that enables individuals to receive professional services and support at a distance” [20 p.2953]. This may involve a Real-Time or Store-and-Forward methods. Real-Time interaction allows patient to communicate in real time with a health care provider. Videoconferencing is viewed a main form of Real-Time communication for telehealth programs. While, Store-and-Forward interaction does not depend on the concurrent presence of parties (e.g. healthcare provider and patient) [21].

Telehealth may have advantages over traditional face-to-face approaches, including improving access to healthcare services especially for individuals and families living in rural and underserved areas [22]. There is growing evidence to suggest that telehealth approaches could decrease providers’ and patients’ costs (e.g. travel time, transportation expenses, missed work) and increase coverage area to providers [23, 24, 25]. Users of such programs can also interact directly with clinicians as well as with instructional content via video, email and apps systems [26, 27]. By offering families with a chance to play an important role in the child’s development, telehealth technology could expedite diagnostic process and early intervention services [26, 28]. However, limited access to the required technologies such as computers and mobile phones could hamper the widespread use of telehealth [29]. In prior telehealth evaluations for children with ASD, parents required good internet access [20, 26, 30]. Such access could be challenging for some communities. Especially, individuals in rural and underserved areas may be disadvantaged by lack of mobile coverage [31]. Slow internet speed, poor quality and unreliable connectivity problems may result in frustration and unwillingness to use telehealth approaches among families and adults with ASD [29]. Some families have concerns that children may change their behaviours if they know they are observed distantly [28, 32]. A further challenge to the use of telehealth seems to be lack of skills and confidence in using technology among some individuals [31]. Reluctance of service providers is also considered as one of the barriers. Limited experience with remote communication systems and concerns about interactions with adults, children and their families have been revealed as potential reasons for their reluctance [32, 33].

The previous literature reported that telehealth is currently used by numerous clinical applications with positive results, such as tele-radiology [34], tele-cardiology [35], and looking into the condition of home-based patients with diabetes and hypertension [21] as well as tele-mental health services [36]. Prior studies also showed that telehealth may be a promising means to facilitate providing a diagnosis in different conditions. For instance, in establishing a diagnosis of dementia, it has been found that telehealth was as accurate as a face to face clinical examination with a high degree of satisfaction correlated with using telehealth [37]. The research also demonstrates that using telemedicine for a congenital heart disease diagnosis is both accurate and safe [38]. Furthermore, telehealth using slit lamp images was suggested to be reliable and accurate method for diagnosis eye problems in accident and emergency departments [39].

Because of the benefits these systems deliver, the notion was moved to the world of ASD [40]. The position of telehealth in the field of ASD is small but growing. Telehealth approaches have recently been explored as a way of supporting the delivery of a range of services for people with ASD and their families. Telehealth might be used to improve individuals’ access to behavioural intervention services. Several studies used telehealth to coach parents of children with ASD in order to conduct behavioural assessments such as functional analyses (FA), functional communication training (FCT) via the use of videoconferencing, either parents and their child located at regional clinic, home or school [41–47]. The findings suggested that parents can successfully conduct FA and FCT through telehealth when behaviour analyst offer consultation remotely. It has also been found reductions in problem behaviour of children. For example, Wacker et al. [46] found the average reduction in problem behaviour was 93.5%. Similarly, Lindgren et al. [41] revealed that problem behaviour was decreased by an average of 90%. It has also been demonstrated the preliminary efficacy of telehealth-delivered parent mediated interventions that are intended to increase parent knowledge and the use of ASD behavioural intervention strategies with the child in their daily life. Several studies suggested that use of telehealth program, combining web-based instructional content with weekly video-conferencing coaching sessions may support parental learning and improve child’s social communication skills [20, 26, 30, 48, 49]. Parents also indicated that such systems were effective, acceptable and useable.

Findings from these studies provide initial evidence for the feasibility, acceptability and effectiveness of telehealth technologies to serve as models for delivering parental training to support conducting the behavioural interventions and helping parents understand and use intervention practices in their daily interaction with children. Given this success, it is possible that telehealth may also be useful in improving the diagnostic pathway of ASD.

So far, very limited research has been conducted in the literature regarding the use of telehealth in ASD. For example, a review by Boisvert et al. [50] included only the studies that used telepractice directly to individuals with ASD, but did not include studies that involved others such as parents of children with ASD. Other previous reviews [51, 52] have focused on studies that centred around the use of telehealth and Internet-based interventions for parental or caregiver training purposes. However, no previous literature review has examined specifically the use of telehealth to support ASD diagnostic assessment. Our review aims to fill this gap and extends prior work by providing a scoping review to examine which telehealth approaches have been used in the diagnosis and assessment of ASD in children and adults, whether they are feasible and acceptable, and how they compare with face-to-face diagnosis and assessment methods. A scoping review was conducted to broadly find out what is known about using telehealth for ASD diagnostic assessment. Contrary to a systematic review which mainly focuses on identifying and appraising the quality of the evidence that is relevant to a particular question/questions [53], a scoping review investigates an area of literature more broadly regardless of methodological quality with intention of examining the extent, nature, and range of research activity [53, 54].

## Method

The PRISMA extension for scoping reviews (PRISMA-ScR) checklist was used to ascertain the key items to report for a scoping review (see S1 Table) [55]. The scoping review was not registred previously.

### Search strategy

The following electronic bibliographic databases were used: MEDLINE, CINAHL Plus with Full text, Business Sources Complete, Web of Science, Scopus and PsycINFO and trail and systematic review databases including Cochrane Library, Health Technology Assessment, Database of Abstracts and Reviews of Effectiveness and NHS Economic Evaluation. Online sources searched included guidelines, government health sites, and ASD related associations. Searches were limited to the English Language from January 2000 to December 2019 inclusive.

The rationale for not including studies prior to 2000 is that telehealth technology was not involved in the ASD field until 2004 [40], however the literature search was conducted from January 2000 to make sure that there is no study that could be consistent with our inclusion criteria. The terms for autism were combined with terms for telehealth to identify eligible studies (see Box 1 an example of a full search in Medline).

**Box 1. Example of search strategy used in MEDLINE database.**

“Autism Spectrum Disorder” OR "Autistic Disorder" OR autis* OR asperger* OR ASD OR neurodevelopmental disabilit* OR developmental disorder* OR Pervasive developmental disorder

AND

telemedicine OR telehealth OR telemonitoring OR ehealth OR mhealth OR m-health OR Telepractice OR Teletherapy OR Telecare OR Telediagnostic OR Teleconference OR Teleassessment OR videoconf* OR mobile tech* OR mobile app* OR web portal OR internet OR web-based OR web-delivered OR Web conference OR online OR Skypeß OR iChatß

AND

diagnos* OR screen* OR assess* OR identif*

Limits: from January 2000 to December 2019, English Language.

Results: 774 studies

### Inclusion and exclusion criteria

Inclusion criteria specified that studies should involve telehealth technology and should report outcomes (not merely describe a service). We define the telehealth technology as any technology that provides ASD diagnostic assessment services at a distance. In respect of the outcomes, as this review is comprehensive, we are considering all of the outcomes that are reported when telehealth has been used in the diagnosis and assessment of ASD in children and adults, e.g. the feasibility and acceptability of telehealth approaches in the diagnosis and assessment of ASD, as well as the accuracy of remote diagnosis compared with in-person methods. As this is a developing field, we included all study designs to fully understand the current state of the literature. Only primary studies were included. The study population was anyone going through an ASD diagnosis process with any kind of healthcare practitioner. Any study that considered only technical issues, editorials or opinions or those concerned with medical education were excluded. In studies of children (younger than 18 years old), information was usually reported by parents/carers, and in studies of adults (18 years old and older), the information could be given by the adults themselves or their carers.

### Data extraction and synthesis

Articles, abstracts or titles identified from the search strategy were reviewed by MA to determine if they met the inclusion criteria. In cases where the eligibility of the study was unclear, it was reviewed by a second reviewer (SB or JC). The full texts were retrieved and reviewed in full by two independent researchers (MA, SB or JC). There was no disagreement in terms of agreement and a third person did not need to adjudicate. Author, year of publication, country, participant characteristics, study design, technology utilised, services delivered, and outcomes were extracted for each paper using a standard form in Excel.

### Quality assessment

Independent assessment of the methodological quality was conducted by two authors (MA, SB or JC) for included studies. In case of differences, consensus was reached by discussion or through consulting (SB or JC). The National Heart, Lung, and Blood Institute (NHLBI) Quality Assessment for Observational Cohort and Cross-Sectional Studies tool [56] was used for five studies [9, 59, 60, 61, 63]. The items of the tool were evaluated by using “yes”, “no”, “not applicable”, “cannot determine”, or “not reported”. This was used to guide the overall rating for the quality of each study as “good”, “fair”, or “poor”. The Diagnostic research Critical Appraisal Skills Program (CASP) tool [57] was also used for four studies [15, 22, 58, 62] and Qualitative research Critical Appraisal Skills Program (CASP) tool [57] for one study [28]. The items of the tools were assessed by using “yes”, “no”, or “cannot tell”. As there are no quality scores in CASP tool, we have decided to use the same quality scores of NHILBI quality assessment tools (“good”, “fair”, or “poor”) [56] for each study.

## Results

### Study characteristics

**Fig 1:**
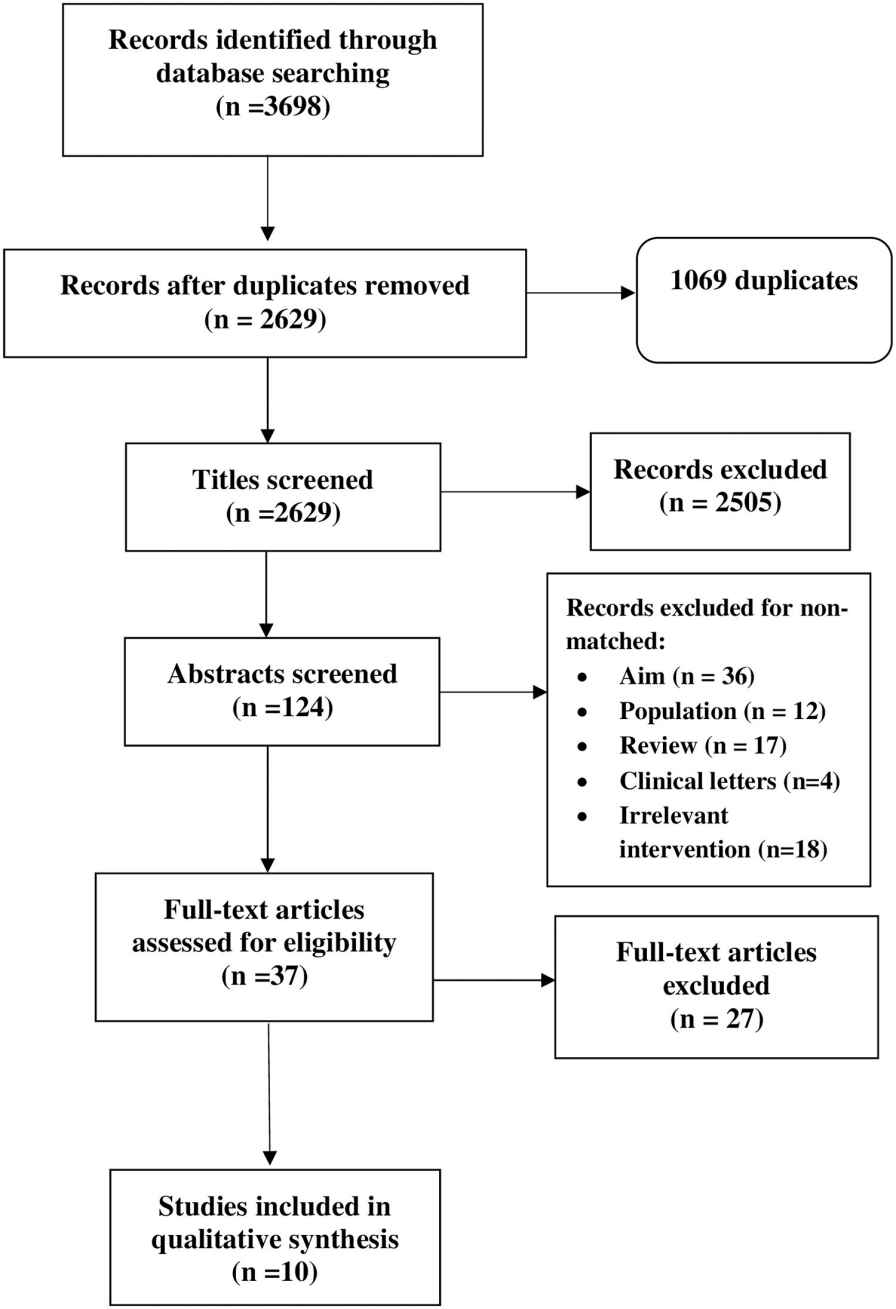
Flowchart of study selection process.

A total of 3698 articles were identified using the search strategy, of which 124 were selected for abstract screening based on title screening. Of these, 37 were selected for full text review and 10 papers were selected for inclusion in the review and data extraction (Fig 1). The studies that were rejected had mainly examined treatment of ASD using telehealth rather than diagnosis. In addition, studies that were reviews, clinical letters, or including a non ASD population or not using telehealth technologies were rejected. All 10 studies were based in the USA and published between 2013 and 2018 (Table 1). There were 5 independent research teams as 3 papers were by Reese et al. [22, 58, 59], 2 were by Nazeen et al. [9, 28], 2 were with Parmanto and team [60, 61], 2 were with Juarez and team [62, 63] and one study was by Smith et al. [15]. A total of 997 people participated across the 10 papers and of these 629 were parents, 291 were children (with ASD) 33 were adults (with ASD) and 44 were clinicians.

**Table 1:** Summary of study characteristics.

Author, year	Country	Participants Characteristics	Study design	Technology utilized	Service delivered	Outcomes
**Nazneen et al., 2015** [28]	USA	43 parents, 51 target children and their siblings, and 10 diagnosticians. Children were between the ages of 18 months and 6 years, 11 months.	Qualitative study Interview	NODA	Remote ASD diagnosis	Parents and diagnosticians found that NODA is easy to use and effective approach to address the obstacles to autism diagnosis. They reported possible barriers such as privacy concerns and reactivity of behavior.
**Nazneen et al., 2015** [9]	USA	**Stage 1: one-on-one interview:** 7 parents of children with autism and 11 clinicians. **Stage 2: iterative development**: 8 families, 18 children and their siblings **Stage3: in-field evaluation:** 4 families with at least 1 child with previous ASD diagnosis and 1 family with a typically developing child and 2 diagnosticians. Children were between 2 and 6 years of age.	Pilot study Interview, development, and evalution.	NODA	Remote ASD diagnosis	Parents can easily record videos of their children behaviors. The clinical validity ratings of the collected videos were 96%. For 4 out of 5 children were remotely diagnosed by both diagnosticians that matched with the child’s actual diagnosis.
**Smith et al., 2016** [15]	USA	40 families seeking an ASD assessment for their children and 11 families of typically developing children. Children were between the ages of 18 months and 6 years 11 months	Diagnostic accuracy study (In-person assessment/NODA)	NODA	ASD remote diagnosis	There was substantial agreement between NODA and in-person assessment (IPA)
**Reese et al., 2013** [58]	USA	11 children with autism and 10 with an existing diagnosis of developmental delay (n = 21). Ages: 3–5 years old.	Pilot study Diagnostic accuracy study	Video- conferencing	Conducting ASD assessment (ADOS-2 and ADI-R) Diagnosis	No significant difference in the diagnostic accuracy, ratings for ADI-R, ADOS observations, and parent satisfaction between IVC and InP conditions.
**Reese, et al., 2015** [22]	USA	17 families of young children requesting an evaluation for ASD. Ages:2.5–6 years old.	Diagnostic accuracy study	Video-conferencing	Conducting ASD assessment (ADOS-2 and ADI-R) Diagnosis	Results revealed excellent interrater agreement on diagnoses between clinicians in the VC setting and interdisciplinary assessment team.
**Reese, et al., 2015** [59]	USA	370 families	Retrospective study	ISUT	Examining telehealth’s impact on families’ access to diagnostic services	ISUT provided families increased access to diagnostic services.
**Parmanto et al., 2013** [60]	USA	5 clinicians 10 adults with disability Age: 17 years old	Case study	Videoconferencing	Remote assessment of adults with ASD (ADOS Module 4)	The patients were satisfied with the developed telehealth system and provided feedback that it was easy to use
**(Schutte et al., 2015)** [61]	USA	23 adults with an ASD diagnosis. Ages: 22 years old	Within-subject crossover study. Questionnaire.	Versatile and Integrated System for Tele-rehabilitation (VISYTER). It consists of videoconferencing and web portal.	Remote assessment of adults with ASD (ADOS Module 4)	Participant satisfaction was high. It demonstrated that an adult autism assessment can be administered distantly with high levels of reliability.
**Juarez et al., 2018** [62]	USA	**Study1:** 20 children and their caregivers (16 boys, 4 girls) Ages: between 20 and 34 months. **Study2:** 45 children and their caregivers (35 boys, 10 girls) Ages: between 19 and 32 months.	**Study1:** Diagnostic accuracy study **Study2:** Feasibility study	Video-conferencing	ASD remote diagnosis	**Study1:** all of the children (n = 15) who received ASD diagnosis via telehealth were confirmed by face to face assessment. **Study2:** clinicians were satisfied with the telemedicine in 80% of cases (n = 36) but would rather to assess a child in person 24% of the time (n = 11).
**Stainbrook et al., 2018** [63]	USA	63 families	Retrospective study	Video-conferencing	Measuring Telediagnosis impact on referrals	Telemedicine diagnostic consultation may positively impact referrals for diagnostic evaluation

ASD = Autism Spectrum Disorder, USA = United States of America, NODA = Naturalistic Observation Diagnostic Assessment, ADOS = Autism Diagnostic Observation Schedule, ADOS-2 = Autism Diagnostic Observation Schedule, Second Edition, ADI-R = Autism Diagnostic Interview–Revised, IVC = Interactive Videoconferencing, ISUT = Integrated System Using Telemedicine, VISYTER = Versatile and Integrated System for Tele-rehabilitation.

### Quality assessment

Quality assessment tools from the NHLBI and CASP were used to assess the methodological quality of the included studies. The overall quality was rated as good, fair, or poor. Eight studies were rated as “fair” quality [9, 15, 22, 58, 59, 61, 62, 63]. Whereas, two studies were rated as poor-quality assessment [28, 60]. In general, most of the studies had small sample size. There were also issues in some studies related to lack of clarity in research questions and objectives; insufficient details provided about sampling, recruitment strategies and inclusion and exclusion criteria; lack of clarity in the rigor of the data analysis and reporting of ethics (see S2–S4 Tables).

### Telehealth technologies

Regarding the assessed technologies, 7 papers using Real-Time method with 5 of these using Videoconferencing and with 2 of these using the Versatile and Integrated System for Tele-rehabilitation (VISYTER) system, and 3 papers used the Store-and-Forward method particularly the Naturalistic Observation Diagnostic Assessment (NODA) software.

### Outcomes

The outcomes are structured and reported according to the telehealth methods (Real-Time/ Store-and-Forward) as each study had different results.

#### Real-time method

*Videoconferencing*. Videoconferencing software enables the families, medical teams and educational teams to meet and discuss care in real time from multiple locations. Three of the studies were by Reese et al. [22, 58, 59] and this series of studies made a connection via Videoconferencing between the specialist hospital and the local health centre or school. Similarly, two studies [60, 61] used videoconferencing. The three studies by Reese et al. [22, 58, 59] used simple videoconferencing to examine (1) the ability of the clinician to assess autism using videoconferencing, (2) the feasibility of clinicians to coach the patients using videoconferencing to correctly complete the assessment activities with their child and (3) the satisfaction of the families with the telemedicine assessment compared to standard face to face assessment. In terms of diagnostic accuracy, these studies found that the diagnosis assessment agreed with the evaluation team diagnosis; 82.5% (n = 28) of the time (for the face to face assessment) and 86% (n = 24) (for the videoconference assessment) [58, 59]. There was no significant difference in the ratings for the ADI-R and ADOS observations between those conducted face to face and those conducted online [58]. There was also excellent agreement between the clinicians across the diagnostic DSM-5 ASD criteria in person and when using the video conferencing approaches [22]. Therefore, these results indicated that there was no significant difference between the face to face and videoconferencing conditions. The final study by Reese et al. [59] found that access was improved and that there was an increase in the diagnostic services for families living in rural and underserved areas. There were 405 families who had the standard face to face assessment and 370 who had the telemedicine assessment. Only 16% returned the satisfaction survey. The children from the telemedicine method were from a wider area (74 counties compared to 35 in the face to face standard method), were diagnosed 3 years earlier and this method also identified older children who had not been previously diagnosed. The travel costs for those who used the telemedicine system would have been substantially more if they had needed to travel for each visit ($100 average cost if the video conferencing families had needed to travel versus $35 average cost for face to face family). The parents were equally satisfied with the video conferencing and the face to face approaches. Two studies by Juarez et al. [62] and Stainbrook et al. [63] examined the accuracy of diagnosis using telehealth (videoconferencing) compared to the in-person assessment in 20 cases and feedback from both the families and clinicians on the acceptability of telemedicine [62]. Furthermore, the impact that telehealth had on the tertiary care referrals has been examined [63]. In terms of the accuracy of the diagnosis, using remote telemedicine procedures, the clinicians identified 75% of cases (n = 15/20) as having ASD. The clinicians rated themselves as “certain” or “very certain” about the classification for 75% of cases. Nineteen of 20 children were diagnosed with ASD by the in-person clinician. All of the children (100%, n = 15) who received the ASD diagnosis by telehealth were verified by a face to face assessment. However, 20% of cases (n = 4) diagnosed by the in-person assessment were not diagnosed remotely. In the evaluation of the feasibility and acceptability of the ASD diagnosis via telehealth (n = 45 children), the clinicians reported that they were ‘certain’ or ‘very certain’ of their assessments in 86.67% of cases (n = 39). The clinicians were satisfied’ or ‘very satisfied’ with the telemedicine in 80% of cases (n = 36) but they would rather to assess a child using an in-person assessment 24% of the time (n = 11), mainly for children who were then referred for a full evaluation (n = 6). This was because some psychosocial factors such as trauma history, complicated diagnostic profiles or technical issues with the telehealth platform (23.8% audio, 4.8% video quality concerns). However, 98% of caregivers were very satisfied with telemedicine experience. The telehealth assessment saved an average of 3.92 h of the anticipated families’ travel time compared to going to the hospital-based ASD clinic.

The study examining the impact of telehealth (videoconferencing) on tertiary care referrals [63] found that the telehealth diagnostic consultation could positively impact referrals for the diagnostic assessment. Telehealth accelerated access to the diagnostic assessment compared to waiting at the tertiary centre. For example, in 2017, the system referred 323 children for evaluation which had a 15-month waiting time. In contrast, the families waited 11 weeks for a telemedicine visit and families kept more appointments. In addition, families were able to schedule and attend appointments. Most families (56 out of 63) preferred the remote method over travelling to the tertiary centre. The referrals to the centre for a complete evaluation were more aimed at the children with different presentations or it was down to family preference.

*Versatile and Integrated System for Tele-rehabilitation (VISYTER)*. Two studies [60, 61] used VISYTER, which incorporates videoconferencing, layout control, image and video presentations, an electronic scoring system and session recording/archiving. This was developed to support the diagnosis of adolescents and adults with possible ASD. It is an off the shelf hardware and it has its own secure server system. There is one desk at the clinician’s site and the other at the local clinic (so the patient needs to attend the clinic in order to use the system). Two cameras are on the patient’s side to capture their eye contact and hand movements. The clinician can zoom and control the camera and take snap shots.

These two studies [60, 61] examined the use of the VISYTER system for the assessment of adults. The participants were between the ages of 19 to 30; 26 out of 46 met the inclusion/exclusion criteria and of these, 14 used the remote method after the face to face method [61]. Of these, the majority had no preference and found both methods to be equal, 5 preferred face to face and 2 preferred remote. In this study, 75% of 14 were comfortable using the remote system, 78% felt the online system correctly represented their typical behaviours and interactions with others. The results supported using telehealth to conduct ADOS remotely. The majority of observations were comparable. However, there were differences in the “Empathy/Comments on others’ Emotions”, “Hand and Finger and other Complex Mannerisms” and “Asks for Information” which may be harder to assess remotely. In the team’s second study [60], they found that the patients (n = 10) were satisfied with the remote system (average score of 6.5 out of 7) and that it scored high for learnability (6.14 out of 7), interface quality (6.5), interaction quality (6.3), that they were very comfortable with the system (6.5) and that they would use it again (6.17). However, the acceptability of telehealth as a substitute for face-to-face was lower (5.86 out of 7).

#### Store-and-forward method

*Naturalistic Observation Diagnostic Assessment (NODA)*. Three studies (2 by the same author (Nazneen) [9, 15, 28] used the NODA system which includes NODA SmartCapture and NODA connect [28]. NODA SmartCapture enables the parents to upload short videos from their smart phones of their child’s behaviour in specific situations (e.g. family meal, play time with others, playtime alone and parental concerns) and remotely share them with a clinician. The first three situations offered opportunities for the child to show play-based behaviours and social-communication skills. Each situation has instructions that contain recording instructions and a sample video to direct the parents about the environment setup. The medical professionals can send additional recording instructions to the parents and it is all uploaded to a webportal. The developmental history of the child is included in the webportal as well. NODA Connect has a list of tags that allows the clinician to determine the presence or absence of behaviours (e.g. no eye contact). These tags are auto-mapped to the (DSM-5) diagnostic criteria. The assessment can be shared with other clinicians and the diagnostic report can be shared with the parents.

The two studies by Nazneen [9, 28] both evaluate the system for development. They involved interviewing the clinicians and families and an in-field evaluation in order to describe the experience of the clinicians and parents as they use NODA. The NODA development study [9] found that parents reported that it was easy to use with a score of 4 on a 5-point scale. The parents were able to follow the instructions and collected the right videos of the right length; 96% of videos recorded by the parents were reported as being clinically useful according to the clinicians. In total, 10 out of 11 of the assessments were given a diagnostic outcome that matched the child’s previous diagnosis. The experience of the stakeholder’s study [28] reported very positive findings for both the parents and clinicians which highlighted that the videos recorded at home allowed the clinicians to observe the evidence of naturalistic behaviours in children which is not possible in clinical settings and is thus otherwise inaccessible. Clinicians reported that the complete assessment of one child required one hour. They felt that the children with classical ASD symptoms would be easily and successfully diagnosed using NODA but children with different presentations would need an additional assessment. It was suggested that NODA could be an effective early screener in these cases [28]. Cases where NODA would be most useful were identified as being with rural families and divorced or separated families; this is as both parents could upload and share videos separately. Therefore, the papers examining the usability of NODA were positive with 40 out of 44 parents feeling that they could use the system and that it would improve the time to diagnosis.

A study by Smith et al. [15] compared NODA (remote approach) with the gold standard (in person assessment) for 40 families with ASD children and 11 with non-ASD children. There was substantial agreement in the diagnosis, with a diagnostic agreement of 88.2% in the full sample and 85% in the subsample. The sensitivity was 84.5% and the specificity was between 94.4% (full sample) and 85.7% (subsample). The Kappa coefficients for inter-rater reliability showed 85% to 90% accuracy between the raters. Therefore, NODA was felt to be a reliable system that performed well compared to the gold standard. It was reported that NODA is not aimed at removing the need for upcoming assessments but that it is intended to accelerate the pathway to early interventions and treatment.

Collectively, these studies concluded that NODA has the potential to improve the efficiency of the ASD diagnostic process.

## Discussion

The literature review found 10 papers (5 independent research teams) that used telehealth in the diagnosis of ASD. Eight of the studies were rated to be of “fair” quality, whereas two studies were rated “poor” using the NHLBI and CASP assessment tools. Most of the studies had small sample sizes (with the range of samples being 10 to 45) with descriptive analysis including a survey design and qualitative experience of the systems. Thus, future high-quality studies are needed to shed more light on this important area. There were 2 methods of using telehealth in the diagnosis of ASD; (1) Real-Time method e.g. Videoconferencing that enables a range of health professionals in different areas to meet in real time with the family to assess the child or adult, thus eliminating the issues of geographical spread. This means that access to specialists might be better facilitated. (2) A Store-and-Forward method as NODA system to upload videos of child’s behaviours and interactions by parents/carers in order to enable the clinicians making an assessment remotely and sharing with parents/carers or other health professionals. This might allow the observations to be conducted by health professionals in the family home remotely, eliminating the need for the family to travel and also enabling for observations to be made in more naturalistic settings. Nearly all the systems identified are designed for ASD to enable the clinicians to undertake an assessment, e.g. the ability to score or mark videos, the ability to zoom in or out to observe facial expressions. This review of the literature found evidence that telehealth methods allow for collaboration and the sharing of experiences between the family, education, ASD experts and the university medical centres and could be equal to face to face methods in terms of satisfaction for the patient, family and clinician. They enable rural families to be seen at lower cost and reduce the time to diagnosis, particularly for those with more severe autism where there is in good agreement in terms of the diagnosis compared to the face to face methods. The findings from the review suggest that telehealth can potentially improve the efficiency of the diagnosis process for ASD.

All of the studies were from the US. However, implementing telehealth systems may facilitate the ASD assessment and diagnosis in other settings with different healthcare systems. In the UK, the multi-disciplinary team approach is one of the assessment methods used for ASD diagnosis, although it is not available in all areas [3, 64]. Research has shown that it is difficult to get a multi-disciplinary team together, especially when they span different disciplines such as education and health [19]. Therefore, telehealth may provide support and help to a specialist ASD team, allowing them to meet remotely and provide a diagnosis regardless of their locations. Moreover, there are critical consequences of a delay in diagnosis as there has been reported a delay of approximately 3.5 years between seeking help and a confirmed diagnosis of ASD [7]. Telehealth technology may help to provide a timely diagnosis and, although it may not appropriate for complicated cases, it could be used as a screening tool for ASD. Consequently, this may increase the opportunity for early intervention as well as reducing the stress for individuals and their families.

### The strengths, limitations and future direction

The strength of this review was in its comprehensive search strategy, intending to find all published studies regardless of method and research quality. However, there were some inconsistencies in the terminology across the studies and a general lack of precision in the descriptions of the telehealth technologies. Furthermore, this review only included papers that were published or written in English. This field is very new and only early design evaluations of the software and the clinician and family experiences of the system are available. There is a need for future high-quality studies using large sample sizes. More clarity in research questions, adequate details about sampling and inclusion and exclusion criteria, more clarity in the rigor of data analysis and reporting of ethics should be taken in the consideration. However, in this early stage, telehealth appears to offer the potential to improve the time to diagnosis of ASD. To date, the evidence suggests that the Real-Time and Store-and-Forward methods such as Videoconferencing and NODA system: (1) are acceptable to both the families and clinicians; (2) have good diagnostic accuracy; (3) enable families from a wider area to access professionals; (3) reduce costs for accessing care; (4) enable the natural behaviours in the home setting to be observed; and (5) may enable both parents in divorced families to contribute to the diagnostic process. This approach to diagnosis is complementary to other developments in this field such as the DAWBA (Development and Well-Being Assessment), which is a computerised assessment using the Strengths and Difficulties Questionnaire to reduce waiting times and inappropriate referrals [65]. The use of the existing telehealth methods could also greatly improve the validity of diagnosis in trials of interventions for ASD in terms of enabling the same team of health professions to see every patient in the trial regardless of location. Finally, the use of telehealth methods seems to be most helpful for those who have clear ASD characteristics as opposed to those whose characteristics may be borderline, and may be useful for the diagnosis of adults with ASD.

### The implications of the findings for practice

This is the first step in examining the potential of using telehealth in conjunction with the current practices in order to improve the time to diagnosis for people with ASD. Larger studies with a randomised design will be needed to confirm that telehealth can improve the diagnosis times.

## Conclusion

Early evidence about the use of telehealth for ASD diagnosis suggests that the systems available to date are acceptable to the families and clinicians. These systems have the potential to improve the time to diagnosis for the families and the people with ASD but warrant further study.

## Supporting information

S1 Table (PRISMA-ScR) checklist. (PDF)

S2 Table Results of quality assessment tool (from CASP) for diagnostic studies. (PDF)

S3 Table Results of quality assessment tool (from the NHLBI) for observational cohort and cross-sectional studies. (PDF)

S4 Table Results of quality assessment tool (from CASP) for qualitative study. (PDF)
