# Supplementary material for: Use of telehealth for facilitating the diagnostic assessment of Autism Spectrum Disorder (ASD): A scoping review
Source: PLoS One. 2020 Jul 23;15(7):e0236415. doi: 10.1371/journal.pone.0236415 (PMC7377392; doi:10.1371/journal.pone.0236415)
Supplement: S2 Table — (PDF) [file pone.0236415.s002.pdf]

S2 Table 2. Results of quality assessment tool (from CASP) for diagnostic studies.

	Smith et al., 2016 [15]	Reese et al., 2013 [58]	Reese, et al., 2015 [22]	Juarez et al., 2018 [62]
1. Was there a clear question for the study to address?	No	Yes	Yes	Yes
2. Was there a comparison with an appropriate reference standard?	Yes	Yes	Yes	Yes
3. Did all patients get the diagnostic test and reference standard?	Yes	Yes	Yes	Yes
4. Could the results of the test have been influenced by the results of the reference standard?	Yes	No	No	No
5. Is the disease status of the tested population clearly described?	Yes	No	Yes	Yes
6. Were the methods for performing the test described in sufficient detail?	Yes	Yes	Yes	Yes
7. Can the results be applied to your patients/the population of interest?	Yes	Yes	No	No
8. Can the test be applied to your patient or population of interest?	Yes	Yes	Yes	Yes
9. Were all outcomes important to the individual or population considered?	Yes	Yes	Yes	Yes
**Quality score**	Fair	Fair	Fair	Fair

Available at: https://casp-uk.net/casp-tools-checklists/
