# Supplementary material for: Use of telehealth for facilitating the diagnostic assessment of Autism Spectrum Disorder (ASD): A scoping review
Source: PLoS One. 2020 Jul 23;15(7):e0236415. doi: 10.1371/journal.pone.0236415 (PMC7377392; doi:10.1371/journal.pone.0236415)
Supplement: S3 Table — (PDF) [file pone.0236415.s003.pdf]

S3 Table 3. Results of quality assessment tool (from the NHLBI) for observational cohort and cross-sectional studies

	Reese, et al., 2015 [59]	Parmanto et al., 2013 [60]	Stainbrook et al., 2018 [63]	Schutte et al., 2015 [61]	Nazneen et al., 2015 [9]
1. Was the research question or objective in this paper clearly stated?	Yes	No	Yes	Yes	Yes
2. Was the study population clearly specified and defined?	Yes	Yes	CD	Yes	No
3. Was the participation rate of eligible persons at least 50%?	No	NA	CD	Yes	NA
4. Were all the subjects selected or recruited from the same or similar populations (including the same time period)? Were inclusion and exclusion criteria for being in the study prespecified and applied uniformly to all participants?	No	NR	Yes	Yes	NR
5. Was a sample size justification, power description, or variance and effect estimates provided?	Yes	No	Yes	NA	No
6. For the analyses in this paper, were the exposure(s) of interest measured prior to the outcome(s) being measured?	Yes	NA	Yes	Yes	NA
7. Was the timeframe sufficient so that one could reasonably expect to see an association between exposure and outcome if it existed?	Yes	CD	Yes	Yes	CD

8. For exposures that can vary in amount or level, did the study examine different levels of the exposure as related to the outcome (e.g., categories of exposure, or exposure measured as continuous variable)?	NA	No	NA	NA	NA
9. Were the exposure measures (independent variables) clearly defined, valid, reliable, and implemented consistently across all study participants?	Yes	Yes	Yes	Yes	Yes
10. Was the exposure(s) assessed more than once over time?	No	NA	NA	No	NA
11. Were the outcome measures (dependent variables) clearly defined, valid, reliable, and implemented consistently across all study participants?	Yes	Yes	Yes	Yes	Yes
12. Were the outcome assessors blinded to the exposure status of participants?	No	CD	CD	No	Yes
13. Was loss to follow-up after baseline 20% or less?	NA	NA	NA	NA	NA
14. Were key potential confounding variables measured and adjusted statistically for their impact on the relationship between exposure(s) and outcome(s)?	NA	No	CD	NA	NA
**Quality score (Good, Fair, or Poor)**	Fair	Poor	Fair	Fair	Fair

Available at: https://www.nhlbi.nih.gov/health-topics/study-quality-assessment-tools. CD, cannot determine; NA, not applicable; NR, not reported.
