# Supplementary material for: Use of telehealth for facilitating the diagnostic assessment of Autism Spectrum Disorder (ASD): A scoping review
Source: PLoS One. 2020 Jul 23;15(7):e0236415. doi: 10.1371/journal.pone.0236415 (PMC7377392; doi:10.1371/journal.pone.0236415)
Supplement: S4 Table — (PDF) [file pone.0236415.s004.pdf]

S4 Table 4. Results of quality assessment tool (from CASP) for qualitative study.

	**Nazneen et al., 2015 [28]**
1. Was there a clear statement of the aims of the research?	Yes
2. Is a qualitative methodology appropriate?	Yes
3. Was the research design appropriate to address the aims of the research?	Can't tell
4. Was the recruitment strategy appropriate to the aims of the research?	No
5. Was the data collected in a way that addressed the research issue?	No
6. Has the relationship between researcher and participants been adequately considered?	No
7. Have ethical issues been taken into consideration?	No
8. Was the data analysis sufficiently rigorous?	No
9. Is there a clear statement of findings?	Can't tell
**Quality score**	Poor

Available at: https://casp-uk.net/casp-tools-checklists/
